# Comparison of Bolus and Continuous Infusion of Adrenocorticotropic Hormone During Adrenal Vein Sampling

**DOI:** 10.3389/fendo.2021.784706

**Published:** 2021-11-26

**Authors:** Jinbo Hu, Jiangqiong Chen, Qingfeng Cheng, Ying Jing, Jun Yang, Zhipeng Du, Ying Song, Linqiang Ma, Yi Yang, Ting Luo, Yue Wang, Qifu Li, Shumin Yang

**Affiliations:** ^1^ Department of Endocrinology, The First Affiliated Hospital of Chongqing Medical University, Chongqing, China; ^2^ Department of Medicine, Monash University, Clayton, VIC, Australia; ^3^ Centre for Endocrinology and Metabolism, Hudson Institute of Medical Research, Clayton, VIC, Australia

**Keywords:** adrenocorticotropic hormone, adrenal vein sampling, primary aldosteronism, adrenal, hypertension

## Abstract

**Background:**

Adrenocorticotropic hormone (ACTH) is widely used in adrenal vein sampling (AVS) and can be administered as a bolus injection or continuous infusion. The optimal administration method has not been determined. We aimed to compare the effects of ACTH bolus with infusion on cannulation success, lateralization assessment and adverse events (AEs).

**Methods:**

Retrospectively collected data from patients with primary aldosteronism who underwent AVS with ACTH at a tertiary hospital in China. Rate of successful cannulation, lateralization index (LI), complete biochemical remission and AEs related to AVS were analyzed.

**Results:**

The study included 80 patients receiving ACTH bolus and 94 receiving infusions. The rate of successful cannulation was comparable between bolus and infusion groups (75/80, 93.4% vs 88/94, 93.6%). In those with successful cannulation, the bolus group had a higher selectivity index than the infusion group, while LI [6.4(1.8-17.5) vs. 7.6(2.0-27.8), P=0.48] and rate of complete biochemical remission (43/44, 97.7% vs 53/53, 100%, P=0.45) did not significantly differ between the two groups. One in the bolus and one patient in the infusion group had adrenal vein rupture but they recovered with conservative treatment. The bolus group reported more transient AEs such as palpitation (52.9% vs 2.2%) and abdominal discomfort (40.0% vs 2.2%) than the infusion group.

**Conclusions:**

Due to their similar effects on cannulation success and lateralization, but a lower rate of transient AEs in the infusion group, the continuous infusion method should be recommended for ACTH stimulation in AVS.

## Introduction

Primary aldosteronism (PA) is one of the most common causes of secondary hypertension (1). Diagnosis of PA requires screening, confirmation, and subtype differentiation. Current guidelines recommend computed tomography (CT) scanning and adrenal vein sampling (AVS) for the subtyping of PA, primarily to distinguish between unilateral aldosterone-producing adenoma (APA) and bilateral adrenocortical hyperplasia (BAH) (2, 3). Because CT scanning alone has been demonstrated to be misleading in some cases, AVS is considered the gold standard for assessing the lateralization of aldosterone secretion and thereby identifying surgically curable forms of PA (4–6). However, AVS remains markedly under-used due to its technically challenging nature, risk of complications (e.g. adrenal vein rupture) and the lack of a uniformly accepted protocol for its performance. Currently, the methods of AVS vary in different centers, including the use of adrenocorticotropic hormone (ACTH). ACTH stimulated-AVS is practiced by around 50% of expert centers (7) but has been reported to decrease the degree of lateralization and potentially mask unilateral APAs (8, 9). However, the potential benefits of using ACTH include higher rates of successful adrenal vein cannulation (10–12) and reduced impact of the time of AVS on aldosterone and cortisol concentrations especially when AVS is not performed in the early morning. Furthermore, ACTH is necessary for patients with contrast allergy who receive steroid treatment before AVS (13). Recently, a study found that ACTH-stimulated AVS might be useful in PA complicated by cortisol co-secretion (14) which has the potential to affect non-ACTH stimulated AVS results (15–17).

To introduce an additional variation in the AVS protocol, the method of ACTH administration varies in different centers, including bolus injection, continuous infusion and bolus plus continuous infusion (18). Some researchers consider continuous ACTH infusion to be preferable because it does not cause the supraphysiological stimulation of the bolus injection that might increase aldosterone production from the contralateral adrenal gland (13). In addition, as the bolus is usually a large dose delivered in a short time, more adverse events (AEs) might be induced by this method. However, no studies have specifically compared the different methods of ACTH stimulation in terms of AEs.

This study therefore aims to compare the effect of bolus ACTH injection and continuous infusion on the rates of cannulation success, assessment of lateralization together with post-operative cure, as well as AEs associated with AVS.

## Methods

### Participants

This study was retrospectively conducted at the First Affiliated Hospital of Chongqing Medical University in China using the database of the Chongqing Primary Aldosteronism Study (CONPASS) (ClinicalTrials.gov: NCT03224312). The inclusion criteria were: 1) hypertensive patients underwent the screening and confirmatory test of PA and confirmed with PA diagnosis by at least one positive confirmatory test (described as follows); 2) completed AVS with ACTH stimulation. PA patients had missing data of AVS or complicated with autonomous cortisol secretion based on abnormal dexamethasone suppression tests were excluded from the analysis. When analyze the effects of different methods of ACTH administration on lateralization and surgical outcomes, those with unsuccessful cannulation (right and/or left selectivity index<3) were excluded. When analyze the blood pressure, heart rate and AEs during ACTH administration, the patients had missing data were excluded.

The ethics committee of the First Affiliated Hospital of Chongqing Medical University approved the protocol. Informed written consent was obtained from each participant.

### Screening and Confirmatory Tests

The methods of screening and confirmatory tests have been reported before (19–21). For PA screening, treatment with diuretics, including mineralocorticoid receptor antagonists, was withdrawn for at least 4 weeks, and angiotensin-converting enzyme inhibitors, angiotensin-II receptor blockers and β-blockers were stopped for at least 2 weeks. Non-dihydropyridine calcium channel blockers and/or α-adrenergic blockers were allowed for uncontrolled hypertension. Samples for plasma renin concentration (PRC) and plasma aldosterone concentration (PAC) were collected in the morning after participants were out of bed for at least 2 hours and after they have been seated for 15 minutes. The screening test was considered positive when the ARR was ≥2.0 ng·dL−1/μIU·mL−1 (54pmol·L−1/μIU·mL−1).

Patients who tested positive proceeded to the confirmatory tests. For patients who tested negative, if PA was strongly suspected based on young age, hypokalemia, or resistant hypertension, they also proceeded to the confirmatory test. The diagnosis of PA was confirmed if at least one confirmatory test was positive: plasma aldosterone concentration (PAC) is not suppressed to less than 8 ng/dl (220 pmol/l) in the saline infusion test or PAC is not suppressed to less than 11 ng/dl (300 pmol/L) in the captopril challenge test or PAC is not suppressed to less than 6 ng/dl (166 pmol/L) in the fludrocortisone suppression test (2, 19). Patients confirmed with PA underwent thin-slice (1 to 3 mm thick) adrenal computed tomography (CT) scan and for those had willing for surgery, AVS was recommended.

### Adrenal Vein Sampling

AVS was performed in the morning between 08:00 and 12:00 with ACTH stimulation (13, 18). During the AVS procedure, a nurse collected the data on AEs, blood pressure, and heart rate before and after (2 and 30 minutes) administration of ACTH.

From November 2016 to September 2019, ACTH was administered as a bolus injection. With local anesthesia, a 5F sheath was inserted into the right femoral vein followed by catheter insertion in the right and left adrenal veins. At this time, 25IU ACTH (amount to 250μg, Shanghai First Bio-chemical Pharmaceutical Company, H31022101) was injected, and 15 minutes later, blood samples were collected simultaneously from bilateral adrenal veins. Three tubes of blood in each adrenal vein were collected consecutively, and one tube of blood in the inferior vena cava (IVC) was collected immediately after the collection of each side of the adrenal vein blood. The adrenal vein blood samples with the highest level of cortisol were usually used for index calculation. As the AEs were often reported by patients following bolus injections of ACTH, from Oct 2019, ACTH was administered as continuous infusion which was started 30 minutes before sampling and continued throughout the procedure at 5 IU/hr (50μg/hr).

The selectivity index (SI), namely plasma cortisol concentration (PCC) in adrenal vein/PCC in IVC>3, was considered to be successful cannulation. The ratio of PAC : PCC on the side with the higher ratio over the contralateral PAC: PCC ratio is defined as the lateralization index (LI). The cutoff for diagnosing lateralization was defined as LI>4 or LI between 3-4 together with a contralateral suppression (PAC/PCC of nondominant side < PAC/PCC of IVC) (13, 18). The diagnosis of APA required complete biochemical success following adrenalectomy, in accordance with the Primary Aldosteronism Surgery Outcome (PASO) criteria (22).

### Biochemical Measurements

PRC and PAC were measured with automated chemiluminescence immunoassays (LIAISON; DiaSorin, Italy). The intra-and inter-assay coefficients of variation for PRC were from 1.2% to 3.7% and from 2.9% to 12.8%, respectively. The analytical sensitivity was 0.53 mIU/l, and the functional sensitivity was 1.6 mIU/l. The lower limit of detection was 0.5 mIU/l. The PAC assay has a measuring range from 2.2 ng/dl (analytical sensitivity) to 100 ng/dl, with a functional sensitivity of 3 ng/dl. The intra-assay coefficient of variation for PAC was from 2.4% to 4.8% and the inter-assay coefficient of variation was from 4.4% to 6.7%. Quality control was performed every day in the laboratory.

### Statistical Analysis

Data distributions were analyzed with the Kolmogorov-Smirnov test. Normally distributed variables were expressed as the mean ± standard deviation (SD); variables with skewed distributions were expressed as the median (interquartile range); categorical variables were described as a percentage. Variables with skewed distributions were analyzed after natural logarithm transformation. Categorical variables were analyzed by the χ2 test, and quantitative variables were analyzed by Student’s t-test. Comparisons at different time points were made using repeated measures of analysis of variance. SPSS 21 was used for statistical analysis. *P*-values <0.05 (two-tailed) were considered statistically significant.

## Results

### Clinical Characteristics of the Subjects

From Nov 2016 to Jan 2021, a total of 85 patients with PA received an ACTH bolus and 99 received an ACTH infusion. One patient with missing data of AVS and nine PA patients complicated with autonomous cortisol secretion (four in bolus group, five in infusion group) were excluded from the study. Details of the ten patients were provided in the **Supplement Table 1**. Finally, a total of 174 patients with PA, including 80 in the bolus group and 94 in the infusion group, were included in the study. There were no significant differences in age, sex, blood pressure, BMI, concomitant diseases, medications, hypertension duration, serum potassium, PAC and PRC between the two groups (**Table 1**).

**Table 1:** Clinical and biochemical characteristics of study participants.

Parameters		Bolus (n=80)	Infusion (n=94)	*P*
Age (y)		48 (40-55)	51 (45-57)	0.099
Sex (female/%)		44/55.0	39/41.5	0.075
SBP (mmHg)		154 ± 18	152 ± 16	0.545
DBP (mmHg)		93 ± 13	92 ± 11	0.464
BMI (Kg/m^2^)		25.1 ± 3.5	25.1 ± 3.6	0.615
Hypertension duration (month)		84 (42-120)	96 (36-144)	0.555
Complicated with diabetes (n/%)		9/12.2	7/9.3	0.557
Complicated with CVD (n/%)		6/10.5	9/11.9	0.804
History of hypokalemia (n/%)		57/77.0	64/83.1	0.348
Use of antihypertensive agents before screening	0-1 agent (n/%)	26/40.0	19/42.2	0.886
	2-3 agents (n/%)	32/49.2	24/53.3	0.671
	>3 agents (n/%)	7/10.8	2/4.4	0.105
Serum potassium (mmol/l)		3.3 (2.9-3.8)	3.3 (3.1-3.7)	0.663
PAC (ng/dl)		21.9 (16.2-33.5)	23.2 (19.3-31.4)	0.253
PRC (μIU/ml)		2.0 (1.2-6.8)	2.4 (0.9-5.4)	0.861
ARR (ng•dl^-1^/μIU•ml^-1^)		10.3 (5.0-20.2)	10.7 (4.3-23.3)	0.653
APA (n/%)		43/53.8	53/56.4	0.715

SBP, systolic blood pressure; DBP, diastolic blood pressure; PAC, plasma aldosterone concentration; PRC, plasma renin concentration; ARR, aldosterone/renin ratio; APA, aldosterone-producing adenoma; CVD, cardiovascular disease.

### Effects of Different Methods of ACTH Administration on Cannulation Success

**Figure 1:**
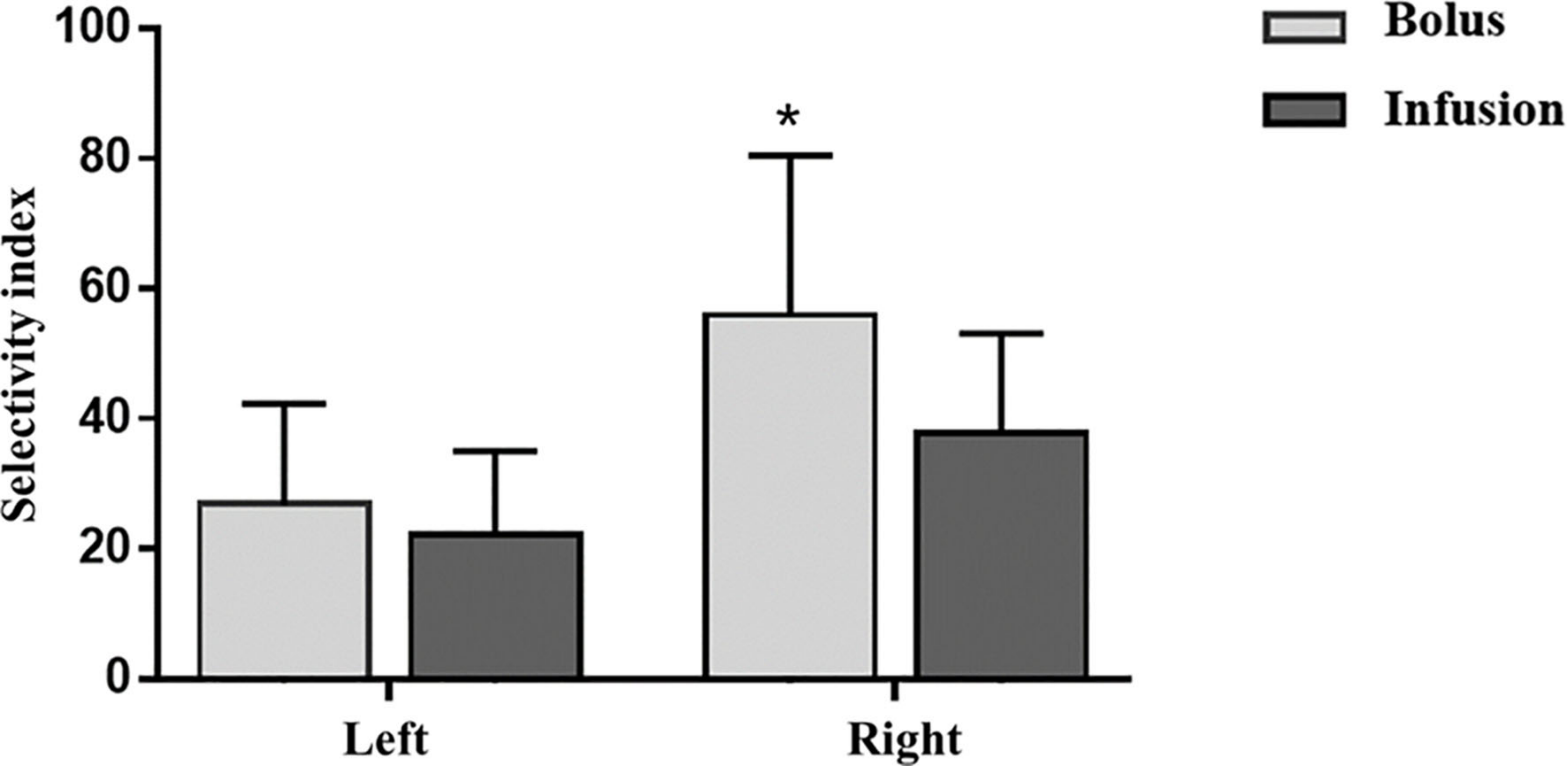
Effects of ACTH infusion and bolus on Selectivity index. Selectivity index is defined as cortisol adrenal vein/cortisol peripheral vein. ACTH, adrenocorticotropic hormone. *P<0.05, bolus vs infusion.

Using SI>3 in both adrenal veins as the criteria for bilateral successful cannulation, five and six patients had unsuccessful cannulation in the bolus and infusion groups respectively, resulting in comparable successful cannulation rates (75/80, 93.4% vs 88/94, 93.6%, P=1.0). In those with successful cannulation, the bolus group had a higher SI in the right adrenal vein and tendency of higher SI in the left adrenal vein when compared with the infusion group (right adrenal vein SI: 56.0(35.5-80.5) vs. 37.8(27.9-53.1), P<0.001; left adrenal vein SI: 26.9(16.7-42.3) vs. 22.1(15.1-35.0), P=0.075; **Figure 1**), but the difference had no impact on the assessment of cannulation success.

### Effects of Different Methods of ACTH Administration on Lateralization

**Figure 2:**
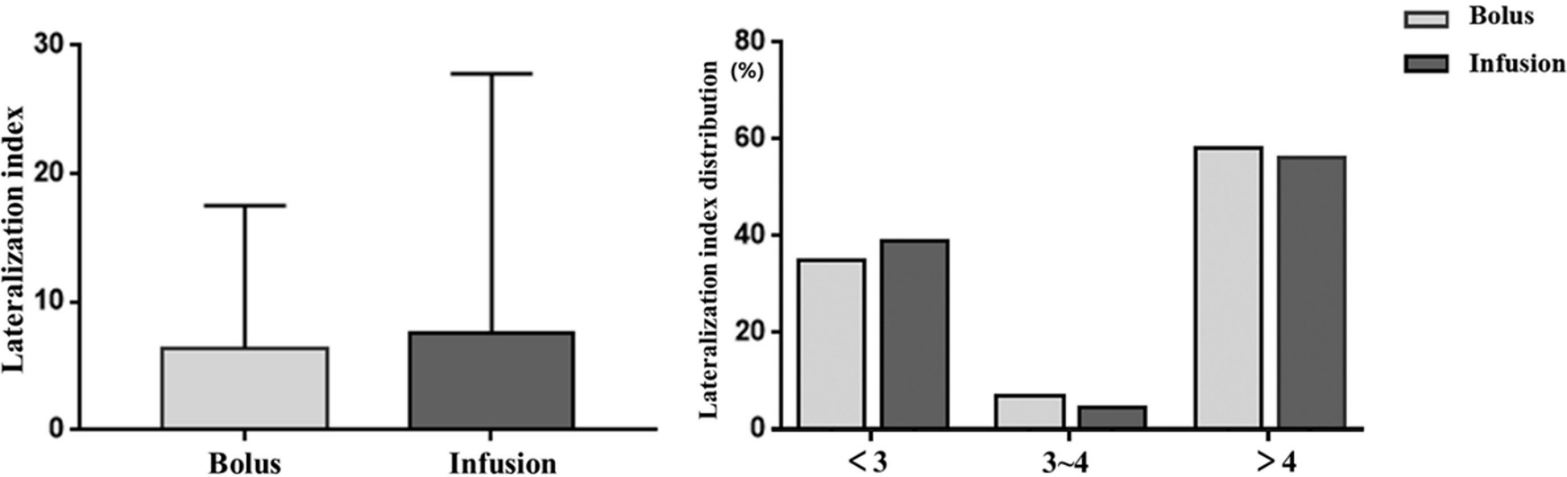
Effects of ACTH infusion and bolus on lateralization index. Lateralization index is defined as aldosterone/cortisol adrenal vein/aldosterone/cortisol contralateral adrenal vein. Left picture: Lateralization index in the subjects; Right picture: Percentage of subjects with different lateralization index. ACTH: adrenocorticotropic hormone.

In those with successful cannulation, PAC, PCC and PAC/PCC ratio in bilateral adrenal veins did not significantly differ between the infusion group and the bolus group (**Table 2**). Compared with the bolus group, the infusion group had higher levels of PAC and PCC in the IVC, but the PAC/PCC ratio in the IVC was not significantly different between the two groups (**Table 2**). Accordingly, the LI was not significantly different between the bolus and infusion groups [6.4(1.8-17.5) vs. 7.6(2.0-27.8), P=0.48]. The number of patients with LI <3, 3-4, >4 in the bolus and infusion group were comparable at 26(35%), 5(7%), 43(57%) and 34(39%), 4(5%), 49(56%), respectively (**Figure 2**).

**Table 2:** Effects of ACTH bolus and infusion on aldosterone and cortisol concentration.

Parameters*	Bolus (n=75)	Infusion (n=88)	*P*
PAC left (ng/dl)	1774.0(598.5-3965.0)	1860.0(508.0-3950.0)	0.941
PAC right (ng/dl)	1610.0(553.0-3625.0)	2290.0(365.0-7100.0)	0.804
PCC left (nmol/l)	11973.0(7442.6-18400.3)	13968.0(8497.0-22305.0)	0.147
PCC right (nmol/l)	25274.0(17355.0-31530.8)	22907.0(17333.0-33997.0)	0.654
PAC/PCC left (ng/dl per nmol/l)	0.2(0.0-0.3)	0.1(0.0-0.2)	0.332
PAC/PCC right (ng/dl per nmol/l)	0.1(0.0-0.2)	0.1(0.0-0.2)	0.984
PAC in IVC (ng/dl)	32.1(22.6-49.6)	43.5(29.2-58.6)	0.002
PCC in IVC (nmol/l)	446.7(390.1-524.1)	630.2(552.9-705.5)	<0.0001
PAC/PCC in IVC (ng/dl per nmol/l)	0.1(0.0-0.1)	0.1(0.0-0.1)	0.897

*The parameters were analyzed only in those with successful canulation. PAC, plasma aldosterone concentration; PCC, plasma cortisol concentration; IVC, inferior vena cava; ACTH, adrenocorticotropic hormone.

### Effects of Different Methods of ACTH Administration on Surgical Outcomes

For those with successful cannulation, 44 and 53 patients in the bolus and infusion group respectively underwent laparoscopic adrenalectomy. Among them, 43 and 53 showed complete biochemical remission. The rate of complete biochemical remission was not significantly different between the bolus and infusion groups (97.7% vs 100%, P=0.454).

### Blood Pressure, Heart Rate, and AEs During ACTH Administration

Twenty-two patients had missing data on blood pressure and/or AEs after ACTH administration (10 in bolus group, 12 in infusion group), therefore, they were excluded from the analysis concerning the vital sign and AEs.

The changes in blood pressure and heart rate during ACTH administration were shown in **Table 3**. There was no significant difference in systolic blood pressure, diastolic blood pressure, and heart rate between the bolus and infusion groups before ACTH administration. Both methods of ACTH administration caused a decrease in systolic blood pressure within two minutes, however the systolic blood pressure was significantly lower in the bolus group (135 ± 20 vs 150 ± 20mmHg, P<0.001). The lowered systolic blood pressure returned to baseline at 30 minutes after ACTH administration in the bolus group but not in the infusion group, resulting in a much higher systolic blood pressure in the bolus group at 30 minutes (157 ± 18 vs 148 ± 20mmHg, P=0.003). The diastolic blood pressure was not significantly different between the two groups before and after ACTH administration.

**Table 3:** Adverse events reported during ACTH administration.

Parameters	Bolus (n = 70)	Infusion (n = 82)	*P*
Palpitation (n/%)	37/52.9	2/2.2	<0.0001
Chest tightness(n/%)	17/24.3	2/2.2	<0.0001
Dyspnea(n/%)	5/7.1	0/0	0.045
Nausea(n/%)	5/7.1	0/0	0.045
Abdominal discomfort(n/%)	28/40.0	2/2.2	<0.0001
Dizziness/headache(n/%)	17/24.3	0/0	<0.0001
Acroanesthesia(n/%)	10/14.3	0/0	0.001
Hot flush(n/%)	16/22.9	0/0	<0.0001

ACTH bolus led to a significantly higher heart rate at 2 minutes when compared to ACTH infusion (85(77-93) vs 69(65-75) beats per min, P<0.001). The heart rate decreased to a level lower than the baseline at 30 min after ACTH bolus, resulting a significantly lower heart rate when compared to patients who had ACTH infusion (62(59-71) vs 68(63-74) per min, P=0.002) (**Table 3**).

One patient in the bolus and one in the infusion group had adrenal vein rupture but they recovered with conservative treatment. Palpitation (52.9%), abdominal discomfort (40.0%), chest tightness (24.3%) and hot flushes (22.9%) were common in the bolus group, while only 2% of subjects in the infusion group reported ACTH-related AEs during AVS (**Table 4**). All these AEs were transient and resolved spontaneously in 30min. None of the patients need special treatment and none of the AVS procedures were cancelled due to these AEs.

**Table 4:** Influence of adrenocorticotropic hormone infusion and bolus on blood pressure and heart rate.

	0min			2min			30min		
	Bolus	Infusion	P	Bolus	Infusion	P	Bolus	Infusion	P
SBP(mmHg)	156 ± 15	156 ± 19	0.878	135 ± 20	150 ± 20	<0.001	157 ± 18	148 ± 20	0.003
DBP(mmHg)	93 ± 12	94 ± 14	0.607	91 ± 12	93 ± 14	0.534	95 ± 12	93 ± 14	0.282
HR(beat per min)	72(67-82)	71(66-79)	0.790	85(77-93)	69(65-75)	<0.001	62(59-71)	68(63-74)	0.002

SBP, systolic blood pressure; DBP, diastolic blood pressure; HR, heart rate.

## Discussion

In patients who seek surgical cure of PA, current guidelines recommend the use of AVS for accurate subtyping (2, 3). ACTH stimulation has been used in AVS by many centers but the ACTH protocols vary between centers. Our study has demonstrated that bolus and continuous infusion of ACTH did not differentially affect the rates of successful cannulation or the assessment of lateralization. However, compared with infusion, bolus injections of ACTH led to more rapid and significant changes in blood pressure and heart rate as well as higher rates of transient AEs, including palpitation and abdominal discomfort, during AVS.

The history of ACTH use in AVS dates back to 1979, when Weinberger et al. introduced continuous infusion of ACTH (5 IU/h) in AVS to reduce errors induced by the episodic production of aldosterone as well as dilution by nonadrenal venous sources (23). Thereafter, ACTH became widely used. A multicenter study of AVS protocols in 24 eligible centers from Asia, Australia, North America, and Europe, found that half of the centers performed AVS with ACTH stimulation (7). The administration protocols included a high dose bolus, continuous infusion and a high dose bolus plus continuous infusion (18).Two previous studies investigated the performance of ACTH infusion compared to bolus injections in patients with PA (8, 10). Silvia et al. investigated the role of continuous ACTH infusion (50μg/h) and bolus (250μg) on the performance and interpretation of AVS in 76 patients with PA (10). The authors reported that LI was not significantly different between methods. In their published data, SI in both adrenal veins seemed to be lower after continuous ACTH infusion than after bolus, but the author did not directly compare the SI between the two protocols as one ACTH protocol was performed in a Japanese center and the other in an Italian center. The other study compared the effects of a high dose (250 μg IV as a bolus, n=47), an intermediate dose (50μg/h, n=14) and a very low dose (250pg IV, n=6) of ACTH on lateralization. Similarly, the authors did not demonstrate a significant difference in the LI between the high dose (bolus) and intermediate dose (infusion). In our study, the LI was not significantly different between the bolus and infusion groups, which confirmed the previous findings.

None of the prior studies evaluated the effect of different methods of ACTH administration on cannulation success, which is the main purpose of using ACTH. Our study reassuringly demonstrated comparable cannulation success between the bolus and infusion groups. While there was a significantly higher SI in the right adrenal vein in patients who received bolus ACTH, the SI in all groups were well above the threshold of 3 for determining successful cannulation and therefore no impact on cannulation success was observed.

Previous studies have reported the effects of ACTH on blood pressure and heart rate (24–28). In the study conducted by Connell et al, they found that muscle injection of ACTH 0.5mg every 12h for five days markedly raised blood pressure in normal man (24). Another study investigated the effect of a continuous 5-day ACTH infusion (40U/24 h) on blood pressure in normotensive and hypertensive subjects also observed increased blood pressure after ACTH infusion (25). Jackson et al. studied the effect of ACTH infusion on blood pressure and heart rate 1- 8h after administration (26). Significant increases in blood pressure and heart rate were observed by 2h but not 1h, and remained generally elevated for the duration of the infusion in their study. The results of the previous studies differ from ours. Notably, the time frame (1 hour-5 days) of the previous studies was much longer than our study (2-30min). Our study illustrated the acute effects of ACTH bolus injection which included an immediate decrease in blood pressure with normalization by 30 minutes, and an increase in heart rate that was accompanied by a drop below baseline after 30 minutes. The decrease in systolic blood pressure was also noted following ACTH infusion, but to a lesser extent, and no change was observed in the heart rate. The mechanism of these acute reactions is unclear. Previous studies reported that ACTH secretion was regulated by carotid body chemoreceptor (29, 30). There is a possibility that ACTH influences the chemoreceptor and related blood pressure regulating areas of the central nervous system. The changes in blood pressure might influence the circulating volume and dilution of adrenal hormones in peripheral blood, which might explain the difference in peripheral cortisol concentration and SI between patients who receive ACTH bolus and infusion.

Up till now, no reported studies have specifically compared the two methods of ACTH stimulation in terms of AEs. As ACTH is mostly used in diagnostic procedures rather than treatment, and tend to be used only for a short period of time, the AEs related to ACTH are rarely investigated in published studies. However, the drug information documents for Cosyntropin (https://www.fda.gov/) as well as Synacthen (http://www.tga.gov.au) mention that tachycardia and bradycardia are the most common AEs of these drugs, in accordance with our findings. The faster changes in blood pressure and heart rate induced by ACTH bolus injection than infusion most likely contribute to the higher rate of AEs reported in the bolus group. In order of frequency, palpitations, abdominal discomfort, chest tightness, dizziness, hot flushes, acroanaesthesia, dyspnea and nausea were reported in up to half of the patients who received ACTH as a bolus injection compared to less than 2% in the group who received ACTH infusion. Although the serious AEs of AVS were not different between the two methods, the transient symptoms caused by bolus would contribute to patient discomfort and are almost entirely preventable using the infusion protocol.

A potential limitation of the present study is that the adrenal and peripheral venous blood before ACTH administration were not collected, therefore, the changes in PAC and PCC induced by different methods of ACTH administration could not be compared directly to the hormone levels before ACTH stimulation. Furthermore, the study is retrospective and the two protocols were applied to different patients over different periods of time. Cannulation success could have improved over time, but were comparable between the two groups mainly because AVS was performed by two dedicated interventional endocrinologists over the entire study period. The comparison of lateralization accuracy of one protocol over the other might be affected by potential differences in the underlying phenotypes of the two cohorts. However, clinical characteristics including age, sex, PAC, PRC and proportion of patients with APA were similar between the two cohorts.

## Perspectives

Guidelines clearly state the importance of AVS in the subtyping of PA and ACTH-stimulated AVS is widely used. Unfortunately, despite being the gold-standard procedure, there are no standardized procedures for AVS. The present study demonstrated that bolus and continuous infusion of ACTH did not differ in their effect on cannulation success or PA subtyping, however, the bolus method caused a higher rate of transient AEs than the infusion method. Therefore, when using ACTH stimulation in AVS, continuous infusion should be recommended. These findings may contribute to the development of a standardized procedure for AVS. Furthermore, the acute decrease in blood pressure following ACTH administration has not been reported before. This phenomenon and its underlying mechanism need to be verified in future studies.

## Data Availability Statement

The original contributions presented in the study are included in the article/**Supplementary Material**. Further inquiries can be directed to the corresponding author.

## Ethics Statement

The CONPASS study was approved by the ethical committee of Chongqing Medical University, and written informed consent was obtained from all patients participating in the study.

## Author Contributions

Conception and design: QL, SY, and JH. Analysis and interpretation of the data: YJ, JC, and QC. Drafting of the article: JH. Critical revision of the article for important intellectual content: JY, and SY. Obtaining of funding: QL, LM, and YS. Administrative, technical, or logistic support: ZD and YS. Collection and assembly of data: TL, YW, LM, and YY. All authors contributed to the article and approved the submitted version.

## Funding

This work is supported by the National Natural Science Foundation of China (81970720, 81870567, 81800731, 81800701, and 82000810); the Science and Technology Research Program of Chongqing Municipal Education Commission (KJZD-K202000401).

## Conflict of Interest

The authors declare that the research was conducted in the absence of any commercial or financial relationships that could be construed as a potential conflict of interest.

## Publisher’s Note

All claims expressed in this article are solely those of the authors and do not necessarily represent those of their affiliated organizations, or those of the publisher, the editors and the reviewers. Any product that may be evaluated in this article, or claim that may be made by its manufacturer, is not guaranteed or endorsed by the publisher.
