# Supplementary material for: Comparison of Bolus and Continuous Infusion of Adrenocorticotropic Hormone During Adrenal Vein Sampling
Source: Front Endocrinol (Lausanne). 2021 Nov 26;12:784706. doi: 10.3389/fendo.2021.784706 (PMC8662304; doi:10.3389/fendo.2021.784706)
Supplement: Supplementary file 1 [file Table_1.doc]

**Supplement Table. Characteristics of the ten patients excluded**

| **No.** | **sex** | **Age**  **(years)** | **Serum k^+^**  **(mmol/l)** | **PAC**  **(ng/dl)** | **PRC**  **(μIU/ml)** | **PAC post-SIT**  **(ng/dl)** | **8am cortisol**  **(mmol/l)** | **8amACTH**  **(pg/ml)** | **cortisol after 1mg DEX**  **(mmol/l)** | **Adrenal CT** | **ACTH methods during AVS** | **SI（left）** | **SI（right）** | **LI** | **Lateralization based on AVS** | **Treatment** | **Complete biochemical success** |
| --- | --- | --- | --- | --- | --- | --- | --- | --- | --- | --- | --- | --- | --- | --- | --- | --- | --- |
| PA1286 | F | 49 | 4.1 | 7.6 | 2.0 | 7.4 | 201.7 | 9.9 | 54.4 | left hyperplasia | bolus | 31.7 | 74.7 | 3.4 | left | medication | NA |
| PA1355 | F | 50 | 3.5 | 20.9 | 3.3 | 9.6 | 270.7 | 13.1 | 128.4 | bilateral nodule | bolus | 23.0 | 30.5 | 9.0 | right | Right adrenalectomy | 1 |
| PA1473 | F | 50 | 2.6 | 34.6 | 2.1 | 37.9 | 277.6 | 11.1 | 105.4 | bilateral nodule | bolus | 11.5 | 3.8 | 2.4 | bilateral | medication | NA |
| PA1568 | M | 57 | 3.2 | 16.3 | 1.3 | 10.6 | 185.6 | 8.0 | 67.7 | left hyperplasia | bolus | 28.0 | 56.0 | 27.6 | left | Left adrenalectomy | 1 |
| PA1729 | M | 48 | 3.6 | 57.8 | 10.9 | 46.6 | 310.6 | 13.6 | 60.9 | left nodule,19mm | infusion | 29.3 | 44.4 | 15.1 | left | Left adrenalectomy | 1 |
| PA1780 | M | 38 | 4 | 13 | 7.2 | 8.8 | 433.5 | 36.2 | 59.0 | bilateral nodule | infusion | 31.6 | 26.2 | 5.8 | right | Right adrenalectomy | 1 |
| PA1844 | F | 58 | 3.4 | 19.2 | 0.5 | 21.5 | 316.2 | 14.2 | 72.5 | bilateral nodule | infusion | 13.2 | 64.4 | 11.3 | right | Right adrenalectomy | 1 |
| PA1935 | M | 57 | 3.7 | 16.8 | 3.3 | 13.4 | 416.5 | 45.8 | 83.6 | right nodule,15mm | infusion | 9.4 | 50.3 | 1.9 | bilateral | medication | NA |
| PA1768 | F | 45 | 1.69 | 56.7 | 0.75 | 100.1 | 318.2 | 30.86 | 143.31 | left nodule,30mm | infusion | 19.5 | failed for cannulation | NA | NA | adrenalectomy | 1 |
| PA1469* | F | 37 | 3.1 | 17.7 | 7.1 | 16.0 | 399.2 | 24.7 | NA | left hyperplasia | bolus | NA | NA | NA | bilateral | medication | NA |

* PA1469 was excluded due to missing data of AVS. The other patients were excluded due to complication with autonomous cortisol secretion based on abnormal dexamethasone suppression tests.

DEX: dexamethasone; NA: not applicable; SI: selectivity index;
